# On Intussusception

**Published:** 1901-09-21

**Authors:** 


### ON INTUSSUSCEPTION.

We last week gave a short epitome of the remarks made by Mr. D'Arcy Power, at Cheltenham, on the subject of intussusception, in which he urged the necessity of early operation in these cases. These remarks, however, were but a part of a general discussion of the subject, the drift of which we now proceed to record. Mr. Bernard Pitts discussing what, as he truly said, was the main question, namely, whether any treatment except immediate operation should be employed, said that in hospital immediate operation gave the best results. The most unsatisfactory feature in attempts at inflation was that one was never sure that one had succeeded in reducing the whole of the intussusception, and it was this uncertainty as to the exact condition of the bowel which made the surgeon prefer to see and handle it. Even when the bowel was outside the abdomen it often required very careful inspection to make sure whether the residual thickening that was left after apparent reduction was due to œdema or to some still unreduced bit of the intussusception, or to some complication. He concluded then by saying that inflation (water pressure seems the most manageable) should only be employed when the case is seen within a few hours of the onset, and is not of a very acute character. In the great majority of hospital cases it is best to open the abdomen at once. Inflation may, however, be tried in certain other cases for the purpose of reducing the main portion of the intussusception and enabling the incision to be made directly over the cæcum. But all this is a surgical and not a medical proceeding. The surgeon should be present and should be prepared to proceed to operation if it fails. Mr. Tubby also was of opinion that the frequent recurrence of intussusception after irrigation or inflation was due to the fact that the condition was not entirely relieved however much it might appear to be so to the hand placed on the abdomen, and in this view he was supported by the great difficulty which was sometimes experienced, even during an abdominal section in completely reducing the last inch or so of the intussusception. He was of opinion that in all but possibly the earliest cases, that is, those of a very few hours' duration, the patient stood a much better chance if abdominal section were performed at once. Mr. Frederick Eve, the president, summed up the matter by saying that the unanimous opinion of the various speakers was against the employment of inflation or injection with the exception of Mr. Pitts, who would employ those measures in cases seen within a few hours of the onset. For his own part he thought that injection or inflation was very rarely efficacious. Of 24 cases so treated at the London Hospital not one was cured by this method alone. Eighteen were subsequently operated on, and six died without operation. Further, injection or inflation was not infrequently followed by an illusory or partial reduction, in which case so-called recurrence of the displacement was inevitable, and the consequent delay in performing the operation led to a fatal result. The whole discussion was of great interest and served in the first place to show the supreme importance of early diagnosis, and, in the next, to illustrate what is every year becoming more clear, namely, that in proportion as one attains such a perfection of technique as to enable one to open the abdomen without fear of thereby doing injury, so one must discard rough and ready methods like injections or inflations and go straight to the injured spot by means of abdominal section. And this not in intussusception alone.

